# Supplementary figures and images for: Uncovering the computational mechanisms underlying many-alternative choice
Source: eLife. 2021 Apr 6;10:e57012. doi: 10.7554/eLife.57012 (PMC8025657; doi:10.7554/eLife.57012)

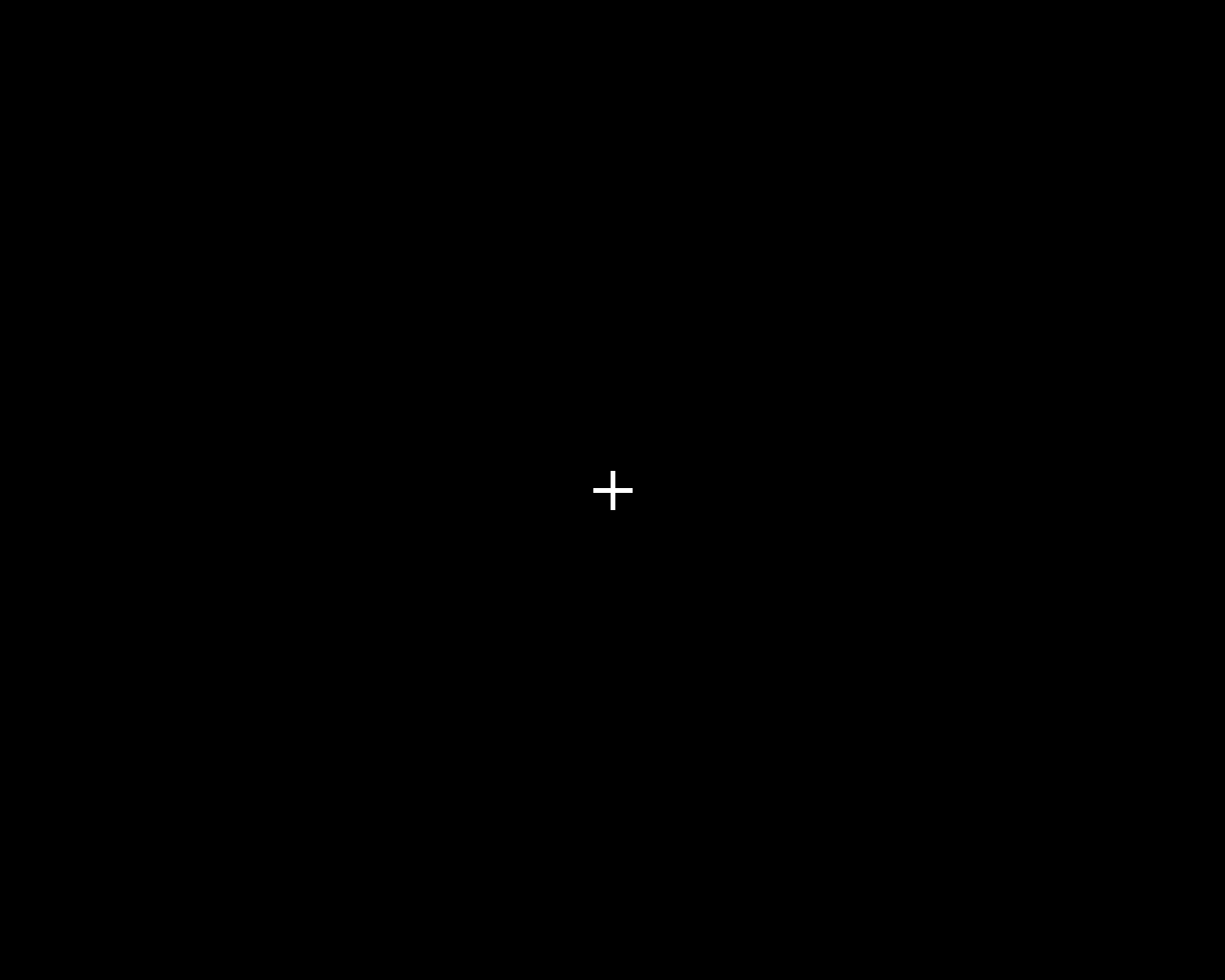

Supplement: Supplementary file 1 [file elife-57012-video1.gif]

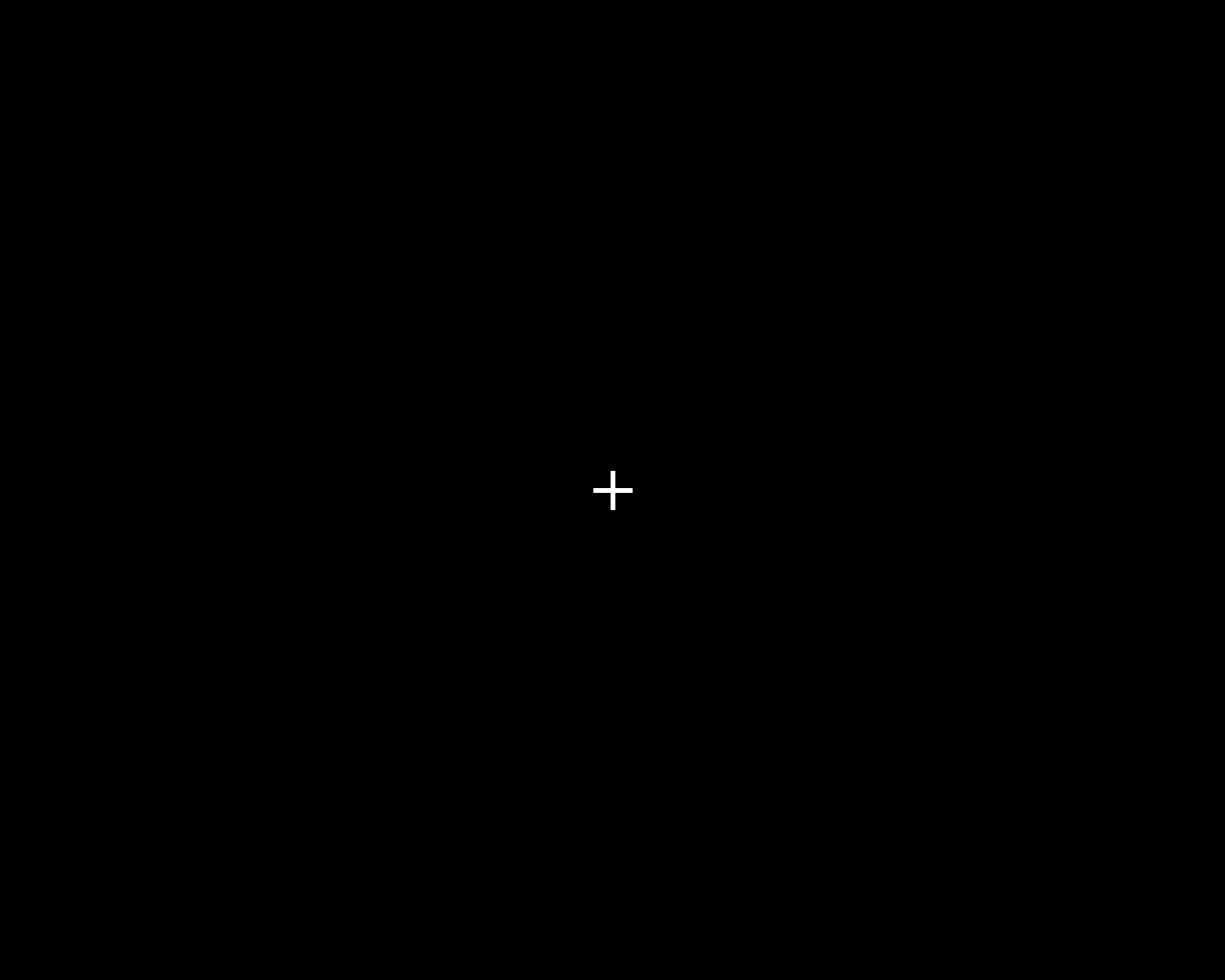

Supplement: Supplementary file 2 [file elife-57012-video2.gif]

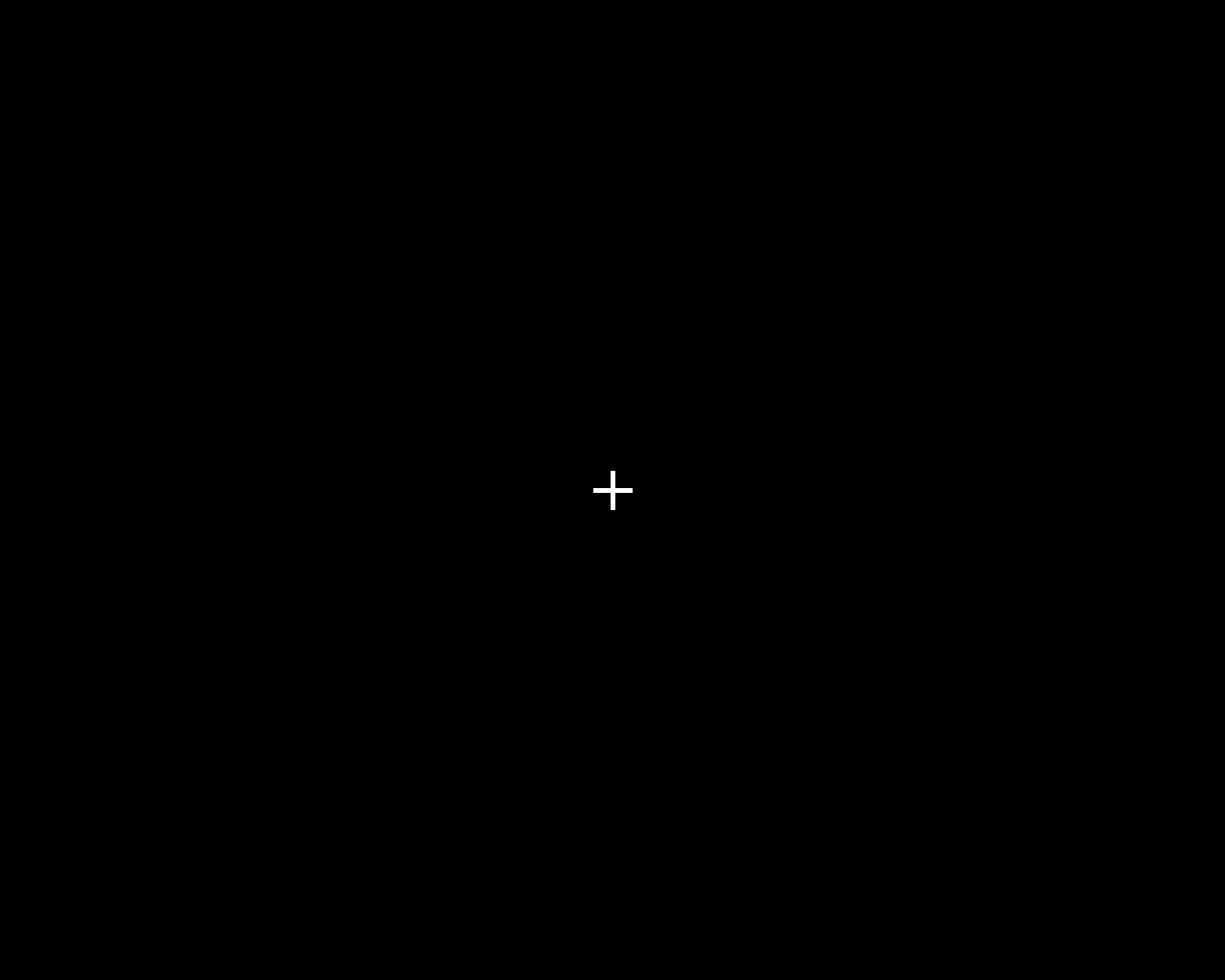

Supplement: Supplementary file 3 [file elife-57012-video3.gif]

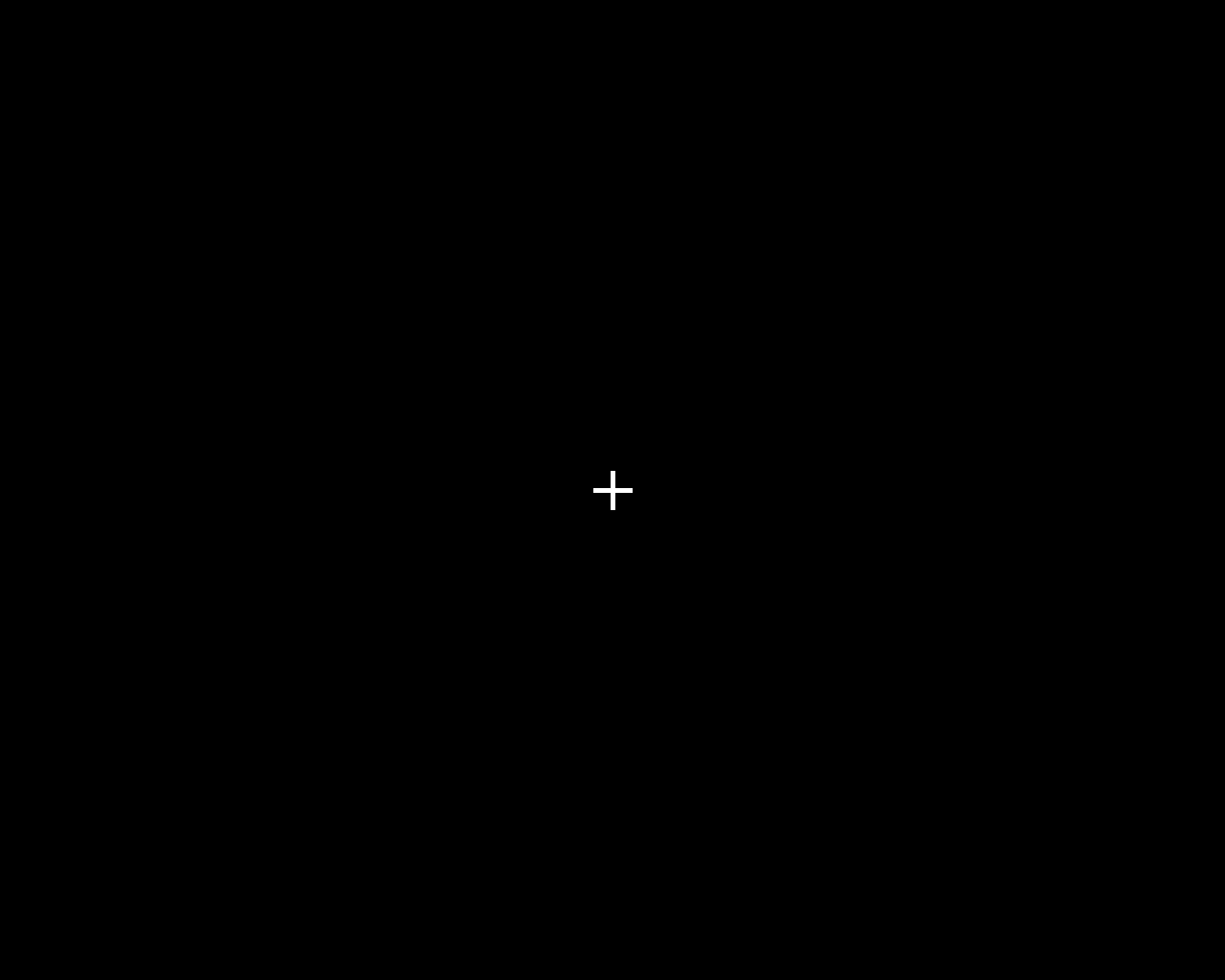

Supplement: Supplementary file 4 [file elife-57012-video4.gif]
