# Supplementary material for: Uncovering the computational mechanisms underlying many-alternative choice
Source: eLife. 2021 Apr 6;10:e57012. doi: 10.7554/eLife.57012 (PMC8025657; doi:10.7554/eLife.57012)
Supplement: Supplementary file 1. — The probabilistic satisficing model has five parameters, determining the additive (ζ) and multiplicative (γ) gaze bias effects on its cached value, the influence of cached value (α) and time (v) on its stopping probability, and the sensitivity of its softmax choice rule (τ). Note that the high mean value of α for the active-gaze variant in the set size with 16 items is driven by one outlier (Figure 5—figure supplement 1D). [file elife-57012-supp1.docx]

| Model | Choice  set size | $\boldsymbol{\gamma}$ | $\boldsymbol{\zeta}$ | $\boldsymbol{v}$ | $\boldsymbol{\alpha}$ | $\boldsymbol{\tau}$ |
| --- | --- | --- | --- | --- | --- | --- |
| *PSM+* | *9* | 0.63 | 1.29 | 1.2e-7 | 1.5e-5 | 3.47 |
| *PSM+* | *16* | 0.53 | 1.73 | 6.6e-8 | 2.5e-5 | 3.53 |
| *PSM+* | *25* | 0.57 | 1.42 | 5.8e-8 | 1.5e-5 | 3.94 |
| *PSM+* | *36* | 0.59 | 1.09 | 4.3e-8 | 1.7e-5 | 4.40 |
| *PSM* | *9* |  |  | 1.2e-7 | 1.5e-5 | 2.51 |
| *PSM* | *16* |  |  | 6.5e-8 | 1.6e-5 | 2.43 |
| *PSM* | *25* |  |  | 5.5e-8 | 1.3e-5 | 2.82 |
| *PSM* | *36* |  |  | 4.0e-8 | 1.2e-5 | 3.06 |

**Supplementary file 1.** Mean parameter estimates of the probabilistic satisficing model with active (PSM+) and passive (PSM) account of gaze in the decision process for each choice set size. The probabilistic satisficing model has five parameters, determining the additive ($\zeta$) and multiplicative ($\gamma$) gaze bias effects on its cached value, the influence of cached value ($\alpha$) and time ($v$) on its stopping probability, and the sensitivity of its softmax choice rule ($\tau$). Note that the high mean value of $\alpha$ for the active-gaze variant in the choice set size with 16 items is driven by one outlier (see Figure 5-figure supplement 1 D).
