# Supplementary material for: Uncovering the computational mechanisms underlying many-alternative choice
Source: eLife. 2021 Apr 6;10:e57012. doi: 10.7554/eLife.57012 (PMC8025657; doi:10.7554/eLife.57012)
Supplement: Supplementary file 2. — The independent evidence accumulation model has four parameters, determining its additive (ζ) and multiplicative (γ) gaze bias effects and its general accumulation speed (v) and noise (σ). [file elife-57012-supp2.docx]

| Model | Choice  set size | $\boldsymbol{\gamma}$ | $\boldsymbol{\zeta}$ | $\boldsymbol{v}$ | $\boldsymbol{\sigma}$ |
| --- | --- | --- | --- | --- | --- |
| *IAM+* | *9* | 0.013 | 0.23 | 1.8e-4 | 0.010 |
| *IAM+* | *16* | 0.005 | 0.20 | 1.7e-4 | 0.011 |
| *IAM+* | *25* | 0.016 | 0.32 | 1.6e-4 | 0.011 |
| *IAM+* | *36* | 0.023 | 0.31 | 1.8e-4 | 0.013 |
| *IAM* | *9* |  |  | 1.9e-5 | 0.014 |
| *IAM* | *16* |  |  | 1.1e-5 | 0.016 |
| *IAM* | *25* |  |  | 1.1e-5 | 0.016 |
| *IAM* | *36* |  |  | 9.0e-6 | 0.019 |

**Supplementary materials 6.** Mean parameter estimates of the independent evidence accumulation model with active (IAM+) and passive (IAM) account of gaze in the decision process for each choice set size. The independent evidence accumulation model has four parameters, determining its additive ($\zeta$) and multiplicative ($\gamma$) gaze bias effects and its general accumulation speed ($v$) and noise ($\sigma$).
