# Supplementary material for: Uncovering the computational mechanisms underlying many-alternative choice
Source: eLife. 2021 Apr 6;10:e57012. doi: 10.7554/eLife.57012 (PMC8025657; doi:10.7554/eLife.57012)
Supplement: Supplementary file 3. — The GLAM variant used in this work has five parameters, determining its additive (ζ) and multiplicative (γ) gaze bias, its general accumulation speed (v) and noise (σ) as well as the sensitivity of the scaling of the relative decision signals (τ). [file elife-57012-supp3.docx]

| Model | Choice  set size | $\boldsymbol{\gamma}$ | $\boldsymbol{\zeta}$ | $\boldsymbol{v}$ | $\boldsymbol{\sigma}$ | $\boldsymbol{\tau}$ |
| --- | --- | --- | --- | --- | --- | --- |
| *GLAM+* | *9* | 0.72 | 2.20 | 3.4e-4 | 0.0067 | 2.63 |
| *GLAM+* | *16* | 0.64 | 2.88 | 2.9e-4 | 0.0057 | 2.51 |
| *GLAM+* | *25* | 0.68 | 2.64 | 2.9e-4 | 0.0050 | 2.32 |
| *GLAM+* | *36* | 0.71 | 2.38 | 2.5e-4 | 0.0047 | 3.02 |
| *GLAM* | *9* |  |  | 3.5e-4 | 0.0069 | 2.24 |
| *GLAM* | *16* |  |  | 2.9e-4 | 0.0058 | 2.17 |
| *GLAM* | *25* |  |  | 2.8e-4 | 0.0052 | 2.36 |
| *GLAM* | *36* |  |  | 2.6e-4 | 0.0047 | 2.77 |

**Supplementary materials 7.** Mean parameter estimates for the gaze-weighted linear accumulator model with active (GLAM+) and passive (GLAM) account of gaze in the decision process for each choice set size. The GLAM variant used in this work has five parameters, determining its additive ($\zeta$) and multiplicative ($\gamma$) gaze bias, its general accumulation speed ($v$) and noise ($\sigma$) as well as the sensitivity of the scaling of the relative decision signals (τ).
